# Bladder training for urinary tract symptoms in Parkinson disease: A randomized controlled trial

**DOI:** 10.1212/WNL.0000000000008931

**Published:** 2020-03-31

**Authors:** Claire McDonald, Jackie Rees, Kristian Winge, Julia L. Newton, David J. Burn

**Affiliations:** From Gateshead Health (C.M.) and Newcastle Upon Tyne Hospitals (J.R., J.L.N.), NHS Foundation Trust; Faculty of Medical Sciences (C.M., J.L.N., D.J.B.), Newcastle University, UK; and Zealand University Hospital (K.W.), Roskilde, Denmark.

## Abstract

**Objective:**

To assess the feasibility and efficacy of bladder training for troublesome lower urinary tract symptoms (LUTS) in Parkinson disease (PD).

**Methods:**

In this single-center, single-blinded, randomized controlled trial, participants with a history of PD and LUTS were randomized to a 12-week bladder training program (BT) or conservative advice (CA). Outcome measures included a 3-day volume frequency diary, International Consultation on Incontinence Questionnaire (ICIQ)–Overactive Bladder Module, and ICIQ—Quality of Life Module. Co–primary endpoints were (1) patient perception of change and (2) change in number of urgency episodes at 12 weeks. Secondary endpoints included change in ICIQ scores, number of micturitions, and volume voided.

**Results:**

Thirty-eight participants were randomized (18 to CA, 20 to BT). Both CA and BT were associated with significant improvements in volume voided, number of micturitions, symptom severity scores, and measures of quality of life (all *p* < 0.05). At 12 weeks, compared to CA, BT was associated with significant superiority on patient perception of improvement (*p* = 0.001), significantly greater reductions in number of voids in 24 hours (mean decrease 2.3 ± 0.8 voids vs 0.3 ± 0.5 [*p* < 0.05]), and greater reductions in interference with daily life (2.1 ± 0.8 point improvement vs 0.3 ± 0.7 point deterioration [*p* < 0.05]). BT was not associated with change in urgency episodes (mean change 2.4 ± 1.5 urgency episodes vs 3.5 ± 1.5 [*p* NS]). At 20 weeks, BT remained associated with greater improvement in interference in daily life. Loss of significance in other measures may reflect loss of power from loss to follow-up.

**Conclusion:**

This controlled trial demonstrated the potential benefits of BT for LUTS in PD.

**Classification of evidence:**

This study provides Class III evidence that for patients with PD and LUTS, BT significantly increased patient perception of improvement but did not significantly reduce urgency episodes.

Lower urinary tract symptoms (LUTS) are common in Parkinson disease (PD) and are associated with poorer quality of life (QOL).^1^ LUTS in PD result from failure of the basal ganglia to suppress micturition. Despite the unique pathology underlying LUTS in PD, trials examining treatments are lacking. Guidelines advocate the empirical use of anticholinergic medication.^2^ However, these drugs are associated with fractures, delirium, and cognitive decline in PD.^3,4^ In the general population without neurologic disease, bladder training (BT) can improve bladder control and continence.^5^ One pilot study examined the efficacy of BT in PD.^6^ While the results were promising, the trial lacked a control group. There is a strong behavioral component to LUTS so studies are susceptible to placebo effects. This controlled study examined the efficacy of BT for LUTS in PD.

## Methods

Participants were recruited from four movement disorder clinics in the North of England. PD was diagnosed by a physician specializing in the diagnosis of PD. All participants reported troublesome LUTS. Exclusion criteria included Montreal Cognitive Assessment score <24, poorly controlled diabetes, indwelling catheter, renal dialysis, cardiac failure requiring diuretics, urinary tract infection, prostate or bladder cancer, uncontrolled bladder outlet obstruction, pelvic organ prolapse, and previous urogynecologic surgery.

### Baseline assessment

Participants completed (1) a 3-day volume/frequency bladder diary; (2) the International Consultation on Incontinence Questionnaire–Overactive Bladder Module (ICIQ-OAB),^7^ a validated means of assessing LUTS severity; and (3) the International Consultation on Incontinence Questionnaire—Quality of Life Module (ICIQ-QOL).^8^

### Randomization

Following baseline assessment, participants were randomized by computer in a 1:1 ratio with stratification by sex (Sealed Envelope Ltd., London, UK) to conservative advice (CA) alone or CA plus BT.

### Interventions

#### Conservative advice

All participants received CA. This comprised instructions to reduce alcohol and caffeine intake and advice regarding the management of constipation and available containment products.

#### Bladder training

Participants randomized to the BT group also underwent a scripted training program. This comprised (1) instructions on urge supersession and distraction techniques, (2) coaching in pelvic floor exercises, (3) a personalized voiding schedule, and (4) a training DVD. Participants in the BT group were asked to return a volume frequency bladder diary every fortnight for 12 weeks. On receipt of the diary, the trainer would review the participant's progress and agree to a new voiding schedule.

### Follow-up

Follow-up assessments were conducted at 12 and 20 weeks by a nurse who was blinded to participant group. All participants were asked to repeat the 3-day volume-frequency diary, ICIQ-OAB, and ICIQ-QOL. At 12 weeks, participants also completed a 10-cm visual analogue scale measuring patient perception of improvement. The scale ranged from 0 (“no improvement”) to 10 cm (“cure”).

### Analysis

As this was a pilot study, a power calculation was not performed. Our initial aim was to recruit 72 participants in 6 months. In keeping with guidelines,^9^ 2 co–primary endpoints were selected: change in number of urgency episodes in 72 hours and patient perception of improvement. Secondary endpoints included change in number of micturitions, number of incontinence episodes, volume voided, and ICIQ-OAB and ICIQ-OAB QOL scores.

### Statistics

Participants with at least 1 follow-up assessment were included in the analysis. Analysis at 20 weeks was performed using the last observation carried forward method. Gaussian data were described using mean and standard error and compared using independent *t* test. Repeated-measures analysis of variance (ANOVA) was used to examine change over time. An interaction between group and time was used to compare BT and CA. Significance was set at *p* < 0.05.

### Standard protocol approvals, regulations, and patient consent

The study was conducted in accordance with Good Clinical Practice Guidelines and was approved by the local Research Ethics Committee and Health Regulatory Agency. Participants provided written informed consent. ISRCTN registry number is 132179.

### Data availability

Anonymized study data can be made available to qualified investigators by contacting the first author.

## Results

**Figure 1:**
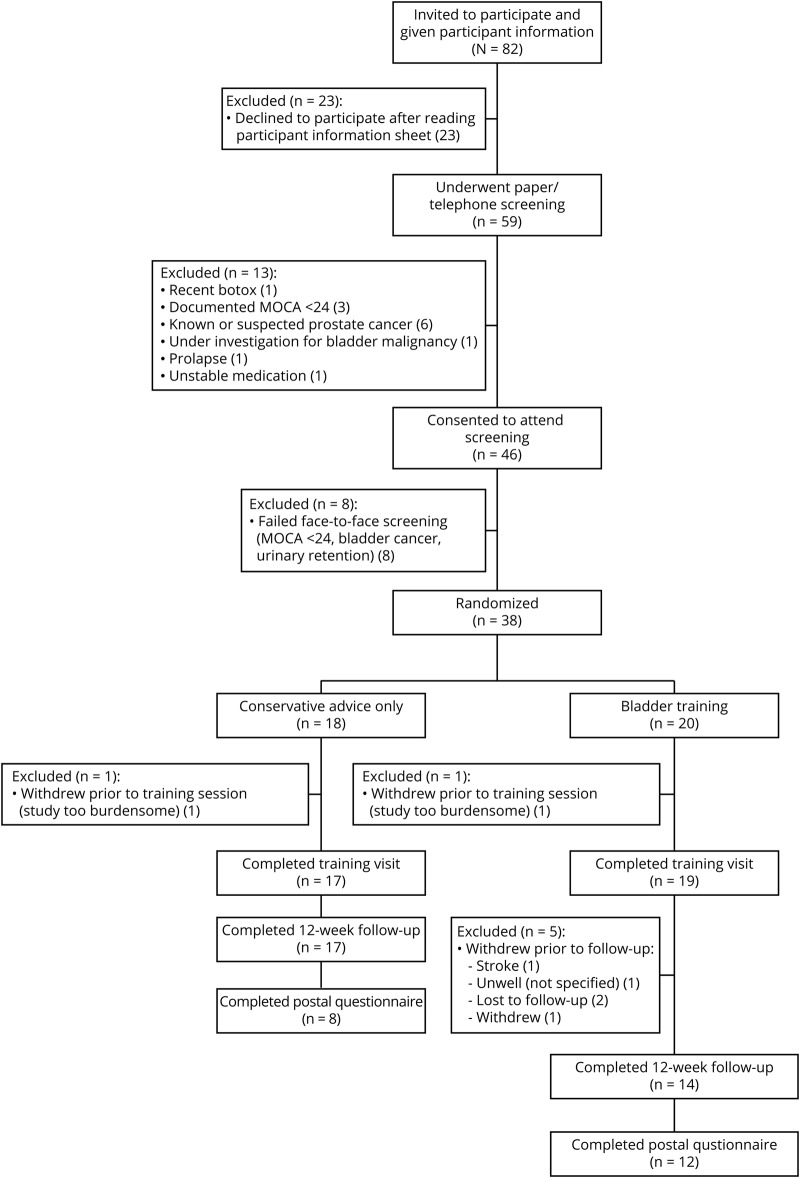
Study recruitment and retention MoCA = Montreal Cognitive Assessment.

Forty-six participants were enrolled in the study between May and December 2017, 38 of whom were randomized. Figure 1 shows recruitment and attrition.

Baseline demographics are shown in table 1. Participants in the BT group were younger than those in the CA group. The groups were similar in terms of PD severity, disease duration, and levodopa equivalent dose. Bladder diary metrics and ICIQ scores were similar at baseline (table 2).

**Table 1:**
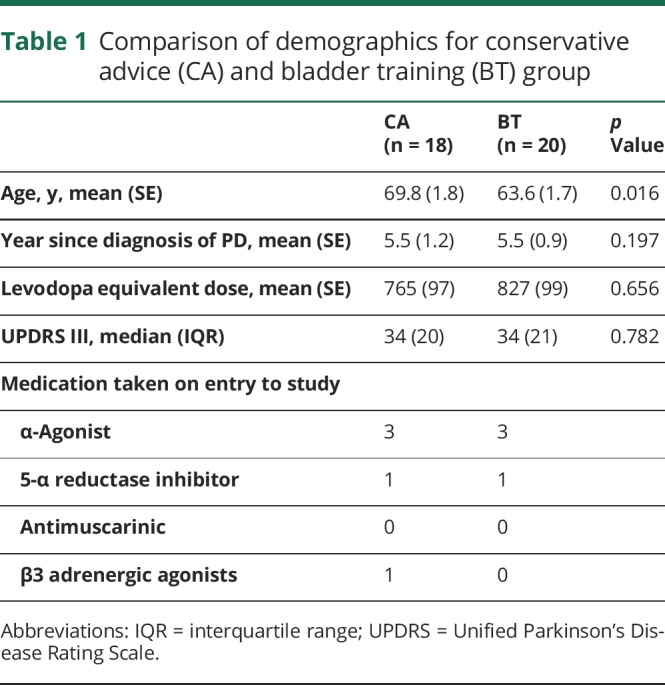
Comparison of demographics for conservative advice (CA) and bladder training (BT) group

**Table 2:**
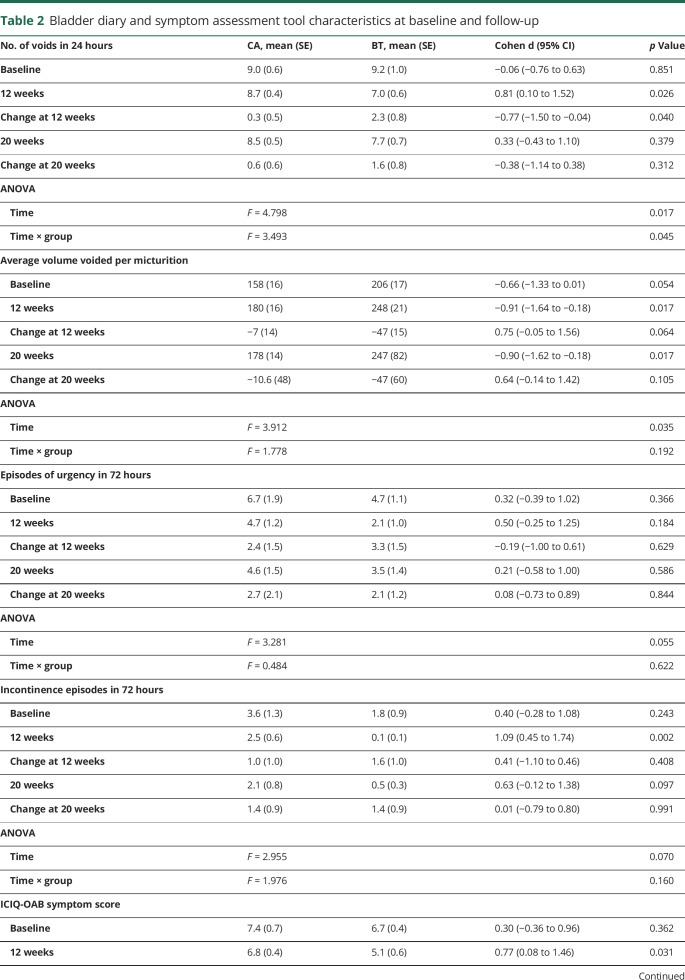

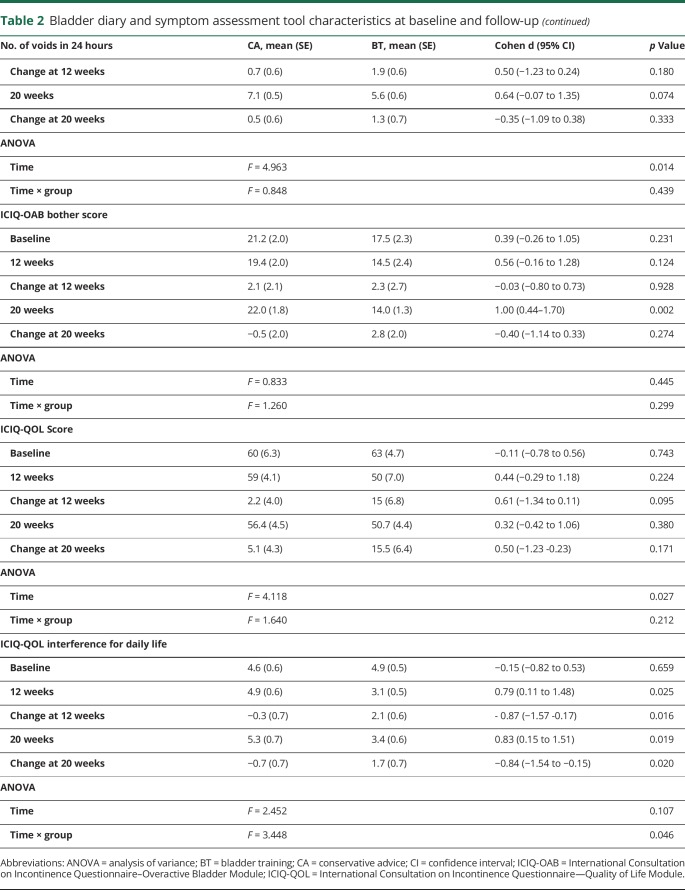
Bladder diary and symptom assessment tool characteristics at baseline and follow-up

### Change in LUTS

ANOVA demonstrated significant reductions in number of voids over the course of the study and a corresponding significant increase in volume voided. There was a trend towards a reduction in number of urgency episodes and incontinence episodes (table 2). ICIQ-OAB scores and QOL scores also showed significant reductions over 20 weeks (table 2).

### BT vs CA

**Figure 2:**
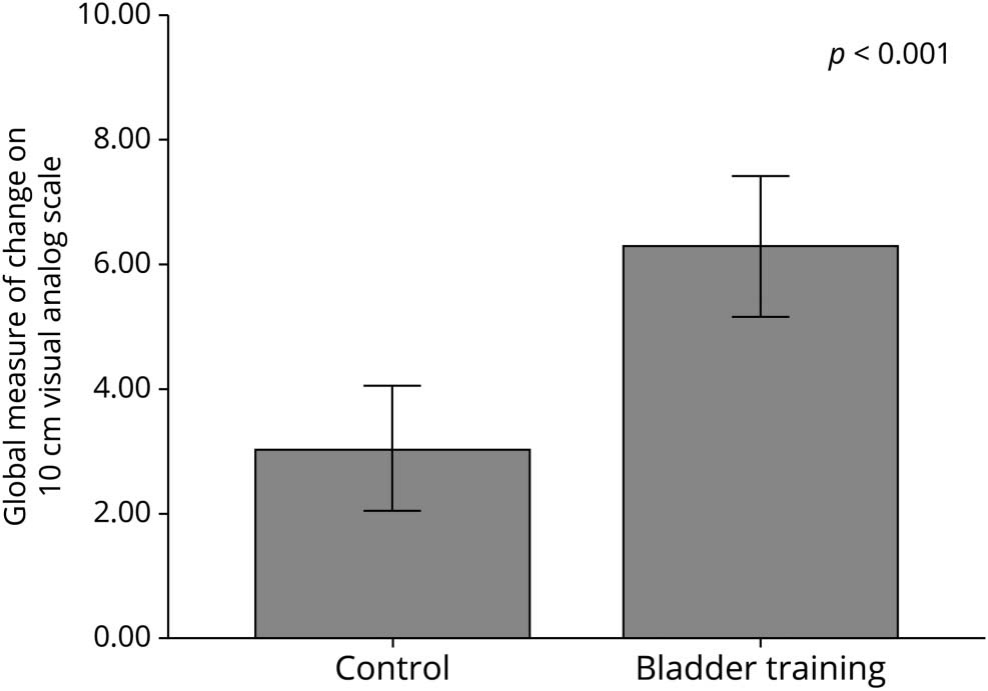
Patient perception of improvement at 12 weeks (10-cm visual analogue scale)

BT was associated with significant superiority for one of the co–primary endpoints: patient perception of improvement (figure 2). At 12 weeks, the BT group reported a statistically significant greater reduction in the number of voids in 24 hours (mean decrease 2.3 voids for BT group vs 0.3 voids for CA group; *p* < 0.05). ANOVA for all 3 assessment periods confirmed an interaction between group and time, suggesting greater efficacy in the BT group. These changes were associated with statistically significant greater improvement in the ICIQ-QOL interference with daily life score among the BT group compared to the CA group. Change in number of urgency episodes or incontinence episodes did not differ between the groups.

At 20 weeks, changes in QOL measures remained consistently better for the BT group than the CA group; however, this was only significant for interference with daily life score. Similarly, volume voided per micturition increased and number of voids in 24 hours decreased at 20 weeks in the BT group compared to the CA group but did not reach statistical significance.

### Concordance and tolerability

A total of 86% of those randomized to BT reported adherence to the program “most or every day” vs 53% of the CA group (*p* = 0.068). Attrition was higher in the BT group (6 participants vs 1 participant; figure 1).

## Discussion

People with PD report a higher prevalence of LUTS than do age-matched controls. LUTS in PD are associated with a poorer quality of life, falls, and admission to long-term care.^1^ Neuroimaging studies demonstrate that micturition is under the control of several cortical and subcortical areas that are common sites for Lewy body pathology, the hallmark of PD.^10^ Animal studies indicate that the net effect of the basal ganglia is to suppress micturition.^1^ In humans, subthalamic deep brain stimulation is associated with reduced prevalence of overactive bladder.^1^ However, few studies have examined interventions for LUTS in PD.

This study demonstrates that both CA and BT may improve LUTS in PD. BT was associated with a significantly greater patient perception of improvement than CA alone. In addition, BT (compared with CA alone) was associated with greater improvements in secondary endpoints including urinary frequency and effect of bladder symptoms on daily life. Urinary urgency did not significantly improve. This may be due to the complex interplay between bladder and motor symptoms. Urgency results from a failure to suppress micturition but may be exacerbated by bradykinesia that results in functional incontinence.

Efficacy of bladder training appeared greatest at 12 weeks. In keeping with other studies, we demonstrated that initial improvements seen on bladder diaries lessen over time.^11^ It should be noted that although not statistically significant at 20 weeks, changes in QOL measures remained consistently better for the BT group than the CA group. The sample size in this pilot study was relatively small and thus the study was likely to be underpowered to detect differences in all endpoints. This was most pronounced at 20 weeks, where numbers were smallest due to attrition.

Only one small study has previously examined the efficacy of BT in PD.^6^ In keeping with our results, Vaughan et al.^6^ demonstrated significant improvements in QOL, symptom score, and bother scores. These investigators used EMG-assisted biofeedback delivered over 5 clinic visits. In our study, the intervention was delivered in a single session without the need for specialist equipment. This study demonstrates that BT could feasibly be delivered by a specialist nurse and our results appear comparable to those obtained by Vaughan et al.^6^

We elected to include patients with troublesome LUTS with and without incontinence. Although LUTS appear to be common in early PD, urinary incontinence appears to be a relatively late presentation.^1^ Our study demonstrated that BT is of help to patients with PD reporting LUTS without incontinence. Furthermore, this study examined BT in PD compared with a control group. Studies of LUTS are highly susceptible to placebo effects. Inclusion of the CA group demonstrated that BT has significant beneficial effects over those derived purely from trial participation.

There are important limitations to our study. Patients in the BT group were slightly younger than those in the CA group. Prevalence of LUTS increases with age but previous studies have indicated that age is not a predictor of outcome from bladder training.^12^ The small sample size meant it was not possible to adjust for differences in baseline characteristics such as age, disease duration, and disease severity or to accommodate for differences during training such as concordance with training. Future fully powered studies would reduce differences between baseline characteristics and should allow for adjustment of these factors in the analysis.

BT is a complex intervention. From this study, it is not possible to ascertain which elements of the program resulted in improved outcomes. The loss of efficacy at 20 weeks may reflect the loss of fortnightly contact from a nurse resulting in loss of concordance with the program, but we cannot say this for certain.

Attrition in the BT group was greater than in the CA group. BT required greater participant commitment; qualitative work is underway to better understand whether this made BT burdensome and to explore potential causes for attrition.

Finally, a higher number of potential participants reported one or more exclusion criteria. Future studies should examine the feasibility, efficacy, and sustainability of behavioral techniques in patients with common comorbidities.

This study demonstrated the potential benefits of BT for LUTS in PD in patients with and without incontinence. Both CA and BT were associated with significant improvements in volume frequency diary and measures of QOL. BT was associated with significantly greater patient perception of improvement, reductions in number of voids in 24 hours, and reductions in interference with daily life than CA alone. Larger studies are needed to confirm these findings.

## Glossary

ANOVA: analysis of variance
BT: bladder training
CA: conservative advice
ICIQ-OAB: International Consultation on Incontinence Questionnaire–Overactive Bladder Module
ICIQ-QOL: International Consultation on Incontinence Questionnaire—Quality of Life Module
LUTS: lower urinary tract symptoms
PD: Parkinson disease
QOL: quality of life
